# Multicompartmental hypothalamic craniopharyngioma resection via expanded endonasal endoscopic transplanum-transtuberculum approach

**DOI:** 10.3171/2026.7.FOCVID26112

**Published:** 2026-10-01

**Authors:** Alessandro De Bonis, Gustavo Passos, Eva M. Wu, Zoukaa Sargi, Carolina G. Benjamin

**Affiliations:** 1 Department of Neurologic Surgery, University of Miami;; 2 Cranial Access, Neuroanatomy and ENT Surgical (CANES) Laboratory, University of Miami; and; 3 Department of Otolaryngology–Head and Neck Surgery, University of Miami, Florida

**Keywords:** expanded endoscopic endonasal approach, transplanum-transtuberculum approach, tubero-infundibular craniopharyngioma, infundibular stalk transection, subpial hypothalamic dissection, safe gross-total resection

## Abstract

Endoscopic endonasal surgery for hypothalamic craniopharyngiomas offers a tailored midline corridor maximizing resection while minimizing brain retraction. Tubero-infundibular lesions present severe technical challenges when engulfing the stalk. In this video, the authors demonstrate an expanded transplanum-transtuberculum approach for a multicompartmental adamantinomatous craniopharyngioma, incorporating detailed anatomical illustrations of the operative corridor. Driven by the case-specific complete tumor engulfment, infundibular stalk transection was the necessary maneuver to progress via tuber cinereum into the third ventricle to separate the lesion from the hypothalamic walls. The deliberate stalk sacrifice proved decisive for achieving safe gross-total resection in the presence of a clear hypothalamic-tumor surgical plane.

The video can be found here: https://stream.cadmore.media/r10.3171/2026.7.FOCVID26112

## Transcript

In this video, we demonstrate a multicompartmental hypothalamic craniopharyngioma resection via an expanded endoscopic endonasal transplanum-transtuberculum approach.

### 0:30 Clinical Presentation.

A 59-year-old woman, previously healthy, presents with a 2-week history of progressive headache and blurry vision. While her right eye functions normally, neuro-ophthalmological testing shows severe visual loss and specific temporal deficits in the left eye. Both her endocrine assessment and neurological exam were still intact.

### 0:48 Neuroimaging Findings.

Contrast-enhanced T1 MRI in the coronal and sagittal planes shows a large, heterogeneously enhancing, multilobulated suprasellar mass, with inferior low signals suggesting calcifications or hemorrhage. The lesion is separate from the pituitary gland, flattening its left superior aspect and completely effacing the stalk, a presentation highly characteristic of a tubero-infundibular craniopharyngioma.

Axial T2-weighted imaging demonstrates the tumor closely abutting, and possibly partially encasing, the left posterior communicating artery. On coronal imaging, the lesion extends superiorly, more pronounced on the left, compressing the undersurface of the optic chiasm with splaying the optic nerves, before extending retrochiasmatically into the third ventricle.^1,2^

### 1:30 Rationale for the Procedure.

Given the massive tumor burden and rapid visual deterioration, early surgical decompression was indicated with the primary goal of maximal safe tumor resection and optic decompression, while preserving hypothalamic structures.^3,4^

### 1:44 Selected Surgical Approach.

The expanded endoscopic endonasal transplanum-transtuberculum approach was selected for its direct midline trajectory and optimal window to the suprasellar and retrochiasmatic spaces, maximizing the safety during the subpial hypothalamic dissection of this left-lateralized tumor.^1,2,5–7^

This corridor also allowed careful assessment of the pituitary stalk and an attempt at preservation, given the patient’s intact preoperative endocrine function. If preservation proved anatomically impossible due to complete radiographic effacement, compromising maximal safe resection, stalk sacrifice was accepted as the necessary trade-off for durable oncological control while preserving hypothalamic integrity.^8^

### 2:23 Alternatives and Why They Were Not Chosen.

In contrast, open transcranial corridors were declined due to the risks of excessive brain retraction, longer recovery, and suboptimal angles for early tumor devascularization and hypothalamic plane dissection. Radiotherapy alone was also deferred given the severe mass effect and acute visual decline.^3,4^

### 2:40 Risk of the Procedure and Its Potential Benefits.

Surgical risks included neurovascular injury, permanent diabetes insipidus, deep hypothalamic damage, and CSF leak. Anticipated benefits were direct chiasm decompression, vision preservation, total resection, and tissue acquisition for molecular profiling.^3,4,9,10^

### 2:56 Description of the Setup.

The patient was positioned supine with the head secured neutrally in a Mayfield frame. Essential equipment includes 0° and 30° endoscopic towers, neuronavigation, needle-tip electrocautery, a high-speed drill, and an abdominal fat harvest kit.

### 3:11 Key Surgical Steps.

The surgical procedure progressed through four main phases.

Stage 1: Nasal phase and wide exposure by the ENT team, sellar floor drilling, tuberculum sellae and planum sphenoidale drilling, medial optic-carotid recess drilling, dural exposure.

Stage 2: Transplanum-transtuberculum dural entry, capsule entry and internal debulking, dissection plane between the tumor and optic chiasm, selective sacrifice of tumor feeders, dissection plane between tumor and posterior communicating artery.

Stage 3: Transection of tumor-engulfed stalk, intraventricular entry via tuber cinereum, dissection plan between tumor and hypothalamus, en bloc tumor removal, and final exposure.

Stage 4: Multilayer skull base reconstruction.

### 4:19 Surgical Procedure: Stage 1.

Following creation of the vascularized nasoseptal flap and exposure of the skull base, a 4-mm diamond burr and a rongeur are utilized to thin and remove the bone overlaying the sella.

The rongeur is then used inferior to the sella floor and clival recess, gaining additional operative freedom beneath the gland. Using both 4-mm and 3-mm diamond burrs, drilling is extended across the tuberculum sellae into the prechiasmatic sulcus and then laterally until both the medial carotid recesses are exposed. Once satisfactory dural exposure of the area is obtained, the dura is coagulated and opened with a horizontal incision across the sphenoid limbus, and followed by vertical incisions toward the tuberculum and superior intercavernous sinus. An additional superolateral oblique dural cut to maximize intradural exposure is also performed.

### 5:12 Surgical Procedure: Stage 2.

With the tumor clearly exposed in the intradural space, the capsule is coagulated and internal debulking is initiated using a combination of an aspirator-dissector and a pituitary punch. A tissue specimen is sent for frozen pathology, confirming craniopharyngioma. Using a Rhoton dissector and sharp microdissection, a superior cleavage plane is gently created above the lesion. The tumor is then removed in a piecemeal fashion with microscissors, allowing further detachment from the chiasm superiorly. During this stage, the superior hypophyseal arteries are identified coursing from the medial internal carotid arteries to the optic chiasm. With gentle maneuvers, these perforating vessels are carefully detached from the tumor capsule and preserved, while tumor feeders arising from it are coagulated and divided. Working underneath the chiasm, dissection is continued along the left side, where the tumor is pushing against the left internal carotid artery. Combining the dissector and the aspirator, the left internal carotid artery is progressively freed from the surrounding tumor and bringing the left posterior communicating artery into view. Piecemeal resection proceeds around the vascular complex until the arteries are fully decompressed.

### 6:29 Surgical Procedure: Stage 3.

Attention is then shifted to the center of the suprasellar compartment, where the infundibular stalk is found to be completely engulfed by tumor, condition that brought us to proceed with stalk transection in order to attempt gross-total resection of the tumor. Working underneath the chiasm, the dissection is continued in the retrochiasmatic space. A cottonoid pledget is placed along the inferior tumor margin, and the tumor is followed and removed piecemeal through the corridor it created into the tuber cinereum, until entry into the third ventricle is confirmed by visualization of the subependymal hypothalamic layer. The primary tumor attachment to the left hypothalamus in the third ventricular floor is identified. Microdissection is meticulously performed to isolate and separate the tumor capsule from the left hypothalamus, in order to carefully avoid any injury. And once the left side is freed, the tumor is gently detached from the right hypothalamus, which it appears to contact rather than infiltrate. And the tumor, now completely free from all attachments, is safely delivered en bloc using a punch.

### 7:35 Surgical Procedure: Stage 4.

The endoscope is advanced into the third ventricle, bringing the bilateral foramina of Monro into view and confirming complete preservation of the hypothalamic plane with no residual tumor. A final inspection also confirms intact internal carotid artery and posterior communicating arteries, alongside a preserved left third cranial nerve. The optic chiasm is fully decompressed, and the bilateral superior hypophyseal arteries remain intact. Reconstruction is then performed using an inlay abdominal fat graft and Surgicel, followed by rotation of the vascularized nasoseptal flap reinforced with Surgicel and fibrin glue.

### 8:14 Postoperative Neuroimaging.

Postoperative contrast-enhanced T1-weighted MRI demonstrates the expected T1-shortening effect of the fat graft within the suprasellar cistern, with no evidence of gross residual disease.

### 8:26 Postoperative Course.

Pathology confirmed an adamantinomatous craniopharyngioma, which was BRAF negative. The patient experienced anticipated postoperative panhypopituitarism and diabetes insipidus, managed successfully with targeted endocrine replacement. She was discharged on postoperative day 7 at her neurological baseline.

### 8:44 Clinical and Imaging Outcome.

At the final 5-year evaluation, the patient demonstrated complete resolution of preoperative headaches and full recovery of her visual deficits, to the extent that corrective lenses were no longer required. She remains stable on long-term endocrine replacement therapy, including desmopressin acetate, hydrocortisone, and levothyroxine.

### 8:58 Five-Year MRI.

Five-year follow-up contrast-enhanced T1-weighted MRI in the sagittal and coronal planes confirms durable, stable tumor control with no evidence of recurrence.

Thank you.

## Acknowledgments

We used Google’s Gemini large language model (May 2026 version) for English-language text editing. Synthetic audio stimuli were generated using ElevenLabs (May 2026 version) via the model "Eleven Multilingual v2" speech synthesis engine. We thoroughly revised, verified, and approved all automated outputs and maintain full accountability for the final content.

## Disclosures

The authors report no conflict of interest concerning the materials or methods used in this study or the findings specified in this publication.

## Author Contributions

Primary surgeon: Benjamin, Sargi. Assistant surgeon: Wu. Editing and drafting the video and abstract: De Bonis, Passos, Wu. Critically revising the work: all authors. Reviewed submitted version of the work: all authors. Approved the final version of the work on behalf of all authors: Benjamin. Supervision: Benjamin, Sargi.

## Supplemental Information

### Patient Informed Consent

The necessary patient informed consent was obtained in this study.
